# Morbidity and mortality of infections in the cirrhotic patients: a US population-based study 

**Published:** 2019

**Authors:** Saad Saleem, Rathnavali Katragadda, Simcha Weissman, Wissam Bleibel

**Affiliations:** 1 *Division of Gastroenterology and Hepatology, Department of Medicine, Mercy St. Vincent Medical Centre, Toledo, Ohio, USA *; 2 *Touro College of Osteopathic Medicine, New York, USA*

**Keywords:** Liver Cirrhosis, infection, morbidity, mortality, hospital costs

## Abstract

**Aim:**

This study aims to investigate the morbidity, mortality, and health care utilization of infection in patients with cirrhosis, with the hope of making clinical recommendations.

**Background:**

The pathophysiology of liver cirrhosis makes patients susceptible to a variety of complications including infection. It contributes to a staggering rate of death and places a tremendous financial burden on the health care system.

**Methods:**

The pathophysiology of liver cirrhosis makes patients susceptible to a variety of complications including infection. It contributes to a staggering rate of death and places a tremendous financial burden on the health care system.

**Results:**

In this cross-sectional study, we queried the National Inpatient Sample (NIS) database for patients discharged from United States (US) hospitals with International classification of diseases (ICD) diagnostic codes consistent with liver cirrhosis, between January 2011 and December 2014. The patients were classified based upon the presence or absence of an infection, as well as their demographics and comorbidities. The data was then analyzed using the IBM SPSS version 25 statistical software.

**Conclusion:**

From 2011 to 2014, 660,727 cirrhotic patients were identified. Of these, 20.6% were found to have an infection. The mortality rate during hospitalization was 4.7% of all cirrhotic patients. The hospital length of stay was significantly longer for the study group than the control group (8.22 days versus 5.11 days) (P <0.0001). Similarly, the mean hospital cost was higher in the study group compared to the control group ($74,729.53 ± $125,963.75 vs. $46,413.32 ± $71,936.50 P< 0.0001).

## Introduction

 Liver cirrhosis results from a liver injury that leads to necroinflammation and irreversible hepatic fibrosis. This pathology makes patients susceptible to a variety of complications, and ultimately a reduced life expectancy. In 2010, cirrhosis was ranked as the eighth leading cause of death in the United States (US) population causing approximately 49,500 deaths (1). 

Significant complications of cirrhosis include gastrointestinal bleeding, infections, hepatic encephalopathy, hepatorenal syndrome, hepatopulmonary syndrome, and ascites. Furthermore, liver cirrhosis is an immunocompromised state, predisposing them to a variety of infections, further increasing mortality about four-fold (2). 

Common infections in cirrhotic patients include spontaneous bacterial peritonitis (SBP) (25%-31%), urinary tract infections (UTI) (20%-25%), pneumonia (15-21%), bacteremia (12%), and soft tissue infection (11%) (3-5). Bacterial infections cause significant morbidity in patients in cirrhosis by leading to liver decompensation, which is found in 20%-60% of hospitalized cirrhotic patients, causing increased mortality rates (6). This study aims to investigate the morbidity and mortality of infection in liver cirrhotic patients, with the hope to guide prophylactic measures. The secondary aim of the study was to evaluate the effect of infections on the hospital stay and hospital cost. 

## Methods

The current study is a cross-sectional study which used the National Inpatient Sample (NIS) database. This database is a part of the Healthcare Cost and Utilization Project (HCUP) sponsored by the Agency for Healthcare Research and Quality in Rockville, MD. The NIS is a database of hospital inpatient stays derived from billing data submitted by hospitals to statewide data organizations across the U.S. The database includes discharge data from more than 1,000 US hospitals, totaling to almost seven million records every year. It uses a redesigned sampling method and contains approximately 20 percent of nonfederal hospitals and is stratified based on geographical location, hospital teaching status, and the number of beds. The NIS contains charge information on all patients, regardless of payer, including individuals covered by Medicare, Medicaid, private insurance, and the uninsured. The NIS database only provides administrative data for analysis; it does not provide laboratory or imaging data. 

**Study sample**

 This **cross-sectional study **included all cirrhotic patients discharged from US hospitals with international classification of Diseases (ICD), Ninth Revision Clinical Modification (ICD-9-CM) diagnostic codes between January 2011 and December 2014. Liver cirrhosis was defined by the following ICD-9 diagnostic codes; 571.0, 571.2, 571.3, 571.5, 571.6, 571.8. The patients were further classified into study group and control group according to the presence of one of these either pneumonia (ICD-9 codes 480, 481,482, 483, 485, 486), or UTI (ICD -9 codes 590, 595) or SBP (789.51 or 789.59) versus absence in their enrolled hospitalization. 

**Statistical analysis**

The study group was compared to control for demographics and other comorbid medical conditions that included age, gender, alcoholism (ICD-9 code 3039.0, 3039.1, 3039.2), hepatocellular carcinoma (HCC) (ICD-9-CM code 155.0), hepatic encephalopathy (HE) (ICD-9-CM code 572.2), gastrointestinal hemorrhage (GI) (ICD-9 578.0, 578.1, 578.9), acute renal failure (ARF) (ICD-9-CM codes 5845, 5849), SBP (ICD-9-CM codes 56723), ascites (ICD-9-CM codes 482) and peptic ulcer bleeding (PUB) (ICD-9-CM codes 531.0, 531.2, 531.4, 531.6, 532.0, 532.2, 532.4, 532.6, 533.0, 533.2, 533.4 and 533.6).

**Variables recorded**

Patient's demographics included age and sex. Length of stay is defined as the number of nights the patient stayed in the hospital. Total hospital charges included the hospital charges for the hospital stay, excluding professional or non-covered charges. The unit of observation is the individual hospital visit and not patient, since one patient may have more than one hospital admission. Age is described with mean and standard deviation; other continuous factors are described with mean and standard; certain factors are described with frequency count and percentages. Age is compared with Student T-test; Mann Whitney Wilcoxon test is used for the other continuous variables. Categorical factors are compared with Chi-square tests. P-values less than 0.05 indicate a statistically significant association. Data were analyzed using the IBM SPSS version 25 statistical software. 

**Table 1:** Comparison of demographics, comorbidities and mortality between study and control group

	Infection and liver cirrhosis (n=136,169)	Non-infection and liver cirrhosis (n=524,558)	P value
Age – n (%)			
30-44 yrs 45-59 yrs 60-74 yrs 75 yrs +	12848 (9.4%) 52811 (38.8%) 48232 (35.4%) 22278 (16.4%)	70715 (13.5%) 233743 (44.6%) 166339 (31.7%) 53761 (10.2%)	0.0001
Gender – n (%) Male Female	63312 (46.5%) 72852 (53.5%) 5 missing	308540 (58.8%) 215957 (41.2%) 61 missing	0.0001
Hepatocellular carcinoma – n (%)	4327 (3.2%)	19705 (3.8%)	0.0001
gastrointestinal hemorrhage – n (%)	8065 (5.9%)	34635 (6.6%)	0.0001
Hepatic encephalopathy – n (%)	25091 (18.4%)	64115 (12.2%)	0.0001
Ascites – n (%)	42585 (31.3%)	127023 (24.2%)	0.0001
Peptic ulcer disease – n (%)	4046 (3%)	18701 (3.6%)	0.0001
Alcohol – n (%)	25806 (19%)	106426 (20.3%)	0.0001
Acute renal failure – n (%)	43607 (32%)	88238 (16.8%)	0.0001
Length of hospital stay (days) mean + SD	8.22+9.47	5.11+5.90	0.0001
Hospital cost ($) mean + SD	$74729.53+125963.75	$46413.32+71936.50	0.0001
Died	9% (12262)	3.6% (19085)	0.0001

**Table 2:** Comparison of healthcare utilization and mortality between cirrhotic patients with and without pneumonia

	Pneumonia	No pneumonia	P value
n (%)	53405 (8.1%)	607,322 (91.9%)	
Length of hospital stay (days) mean + SD	9.45+10.66	5.42+6.37	0.0001
Hospital Costs ($) mean + SD	$93802.54+155546.435	$48594.41+76658.675	0.0001
Mortality	6883 (12.9%)	24464 (4.0%)	0.0001

## Results

In the period of study from 2011 to 2014, 660,727 cirrhotic patients were identified. The population consisted of 371,852 (56.3%) males and 288,809 (43.7%) females. The mean age was 58.5 ± 12.54 years. During the hospitalization, 136,169 (20.6%) cirrhotic patients were found to have pneumonia, or urinary tract infection (UTI) or spontaneous bacterial peritonitis (SBP). The mortality rate during hospitalization was 4.7% for the cirrhotic patients. 

The demographic characteristics, morbidity, and mortality of study and control groups are shown in Table 1. There were more female patients in the study group as compared to the control group (53.5% vs. 41.2%; P < 0.0001). Patients were more aged in the study group than in the control group (75 + years 16.4% vs. 10.2%; P < 0.0001). Patients with infections had an increased risk of morbidity that includes ARF (32% vs. 16.8%; adjusted odds ratio (OR) 2.33; (95% CI 2.23 - 2.36)), HE (18.4% vs. 12.2 %; OR 1.62 (95% CI 1.60-1.65)), ascites (31.3% vs. 24.2%; OR 1.42 (95% CI 1.41-1.44). More patients were noted in the control group than in the study group for the following morbidities: HCC (3.2% vs. 3.8% P< 0.0001), UGIB (5.9% vs. 6.6% P< 0.0001), PUD (3% vs. 3.6% P < 0.0001). 

**Table 3:** Comparison of healthcare utilization and mortality between cirrhotic patients with and without spontaneous bacterial peritonitis (SBP)

	SBP	No SBP	P - value
n (%)	14033 (2.1%)	646694 (97.9%)	0.0001
Length of hospital stay (days) mean + SD	8.55+ 9.37	5.69+6.83	0.0001
Hospital Costs ($) mean + SD	81983.41+146017.983	51615.33+84825.028	0.0001
Mortality	2286 (16.3%)	29061 (4.5%)	0.0001

**Table 4:** Comparison of healthcare utilization and mortality between cirrhotic patients with and without urinary tract infection (UTI)

	UTI	No UTI	P-value
n (%)	79948 (12.1%)	580779 (87.9%)	P < 0.0001
Length of hospital stay (days) mean + SD	7.93+9.508	5.45+ 6.409	P < 0.0001
Hospital Costs ($) mean + SD	68050.20+115003.163	50076.91+81761.152	P < 0.0001
Mortality	5167 (6.5%)	26180 (4.5%)	P < 0.0001

The hospital length of stay was significantly longer for the study group than the control group (8.22 days versus 5.11 days) (P <0.0001). Similarly, the mean hospital cost was higher in the study group compared to the control group ($74,729.53 ± $125,963.75 vs. $46,413.32 ± $71,936.50 P< 0.0001). In the study group, the inpatient mortality rates were 9%; whereas, in the control group inpatient mortality rates were 3.6%. The odds of dying in the hospital were 2.62 times (95% CI: 2.56 to 2.68) greater for study group compared to the control group. 

From 2011 to 2014, the total number of hospital admissions with a diagnosis of pneumonia, SBP and UTI was 8.1%, 2.1% and 12.1%, respectively. The mean aggregated costs of hospitalizations increased by 93% if pneumonia was the diagnosis in cirrhotic patients as compared to without diagnosis of pneumonia ($93802.54 ± $155,546.435 vs. $48,594.41± $76,658.675, P < 0.0001). There was an associated increase of 74% in the average length of hospital stay with the diagnosis of pneumonia (9.45 days vs. 5.42 days P < 0.0001). Mortality was higher in the pneumonia patients as compared to control group (12.9% vs. 4.0%; OR 2.72 (CI 2.65- 2.80) P < 0.0001) (shown in table 2). 

The average length of hospital stay for a cirrhotic patient with SBP was 50% more as compared to without SBP infection (8.55 days vs. 5.69 days respectively, P < 0.0001). The mean hospital charges were increased by 59% in the cirrhotic patients with the diagnosis of SBP ($81983.41 ± $146017.983 vs. $51615.33 ± 84825.028 P<0.0001 respectively). Cirrhotic patients with the diagnosis of SBP had higher mortality (16.3% vs. 4.5%; OR 4.13 (CI 3.95- 4.323) P < 0.0001) (shown in Table 3). 

The average length of hospital stay for a cirrhotic patient with UTI was 7.93 days as compared to 5.45 days in cirrhotic patients without UTI (an increase of 46%, P < 0.0001). The aggregated costs of hospitalizations were significantly higher in UTI than in the non-UTI group ($68050.20±$115003.163 vs. $50076.91± $81761.152 respectively) with an increase of 36% (P<0.0001). Cirrhotic patients with the diagnosis of UTI had 1.46 times higher mortality than without UTI infection (6.5% vs. 4.5%; OR 1.46 (CI 1.42- 1.51) P < 0.0001) (shown in Table 4). Although the frequency of hospital discharges for UTI as a diagnosis was highest among the three infectious diagnoses, pneumonia caused the highest increase in hospital stay and cost as compared to without pneumonia in cirrhotic patients. SBP was associated with the highest mortality among the three infectious processes.

## Discussion

Our data demonstrated that the rate of infection in the cirrhotic population was higher in patients more than 60 years of age. Females had a higher prevalence of infections compared to males. The incidence of infections in the cirrhotic population led to a longer length of hospital stay and hospital cost. Infection in cirrhotic patients increased the inpatient morbidity of HE, ARF, ascites and mortality rates. 

Liver cirrhosis is a disease that has multiple causal factors, the most common being alcoholic liver disease and hepatitis C. The natural history of the disease is complicated by numerous conditions like ascites, hepatic encephalopathy, gastrointestinal bleeding; hepato-pulmonary and hepato-renal syndromes. 

Liver cirrhosis predisposes patients to infections. Numerous pathogenetic mechanisms were thought to be causing infections; the most accepted hypotheses are being decreased body immune defenses, reticuloendothelial dysfunction, loss of normal gut flora (6). The main aim of our research was to study the most common infections in hospitalized patients with liver cirrhosis and its association with various complications during hospital stay.

In our study, the incidence of infection was significantly higher in patients more than 60 years of age as compared to early adulthood indicating that age increases the risk for infections. Infections in liver cirrhosis were found to be higher in females as compared to males similar to the study by Rubin *et al.* but established that cirrhotic complications and related mortality were lesser in females (7).

Among hospitalized patients with liver cirrhosis, 1/5th of them were found to have infections. This incidence was lesser compared to most other research probably because of a large number of populations studied. UTI was the most common infection identified in these patients as with findings of many other study groups (8,9), followed by Pneumonia and spontaneous bacterial peritonitis. We reported that all infections in liver cirrhosis precipitated increased mortality in patients with liver cirrhosis with SBP having the highest risk. This was comparable with other studies on infection-related mortality in patients with cirrhosis (10,11). 

These infections are presumed to cause acute on chronic liver disease (11,12) and complications related to cirrhosis thereby leading to increased morbidity and mortality. This is proven by our study where patients with identified infections in the hospital were found to have a higher association with hepatic encephalopathy and acute renal failure, a result consistent with previous studies (13,14). The incidence of ARF was markedly increased in patients with infections compared to those who did not have infections. ARF was thought to be the most important complication leading to increased mortality (15) as is an acute liver failure (12,15). Hence, the strong association between ARF and infections in cirrhosis patients established in our study emphasizes the importance to prevent renal failure in patients with cirrhosis and infections.

Surprisingly, they did not show increased association with hepatocellular carcinoma, upper GI bleed or peptic ulcer disease as compared to those without infections contradictory to previous findings of increased GI bleed with infections (8). The increased risk of cirrhosis complications in patients with infections was thought to be due to sepsis-related immunological response and hypotension causing tissue damage, multi-organ failure leading to increased mortality (15). 

As much as our study emphasizes the importance of preventing and treating infections earlier, so that it can decrease the complications and mortality in patients with liver cirrhosis; it is also essential to know how much the disease burden affects the health care costs. Hence, we also studied how the co-existence of infections in liver cirrhosis affected the length of stay of patients in the hospital and its effects on hospital charges. Infections increased both the length of stay and hospital charges in patients with cirrhosis. Pneumonia was associated with the maximum increase in the length of stay and hospital costs followed by SBP and UTI. There was no research to our knowledge studying the effect of infections in cirrhosis on health care costs. Although, it was noted that hospital care costs significantly increase in the last year of life in patients with end-stage liver disease as is the length of hospital stay (16,17).

Analysis of health records by Jamil *et al.* concluded that hepatorenal syndrome that was associated with increased hospital stay and health care costs markedly, the most salient inference being increased mortality despite increased health care burden to prevent poor outcomes (18).

To conclude, infections in cirrhosis were strongly associated with end-stage liver disease complications, importantly ARF. It is understood that ARF and hepatorenal syndrome are one of the highest predictors of mortality in patients with cirrhosis and increased health care burden. Appropriate and aggressive infection prevention protocols may be an effective modality to prevent infections, and thereby morbidity and mortality making effective use of health care.
